# Assessing the Fiscal Burden of Overweight and Obesity in Japan through Application of a Public Economic Framework

**DOI:** 10.36469/001c.123991

**Published:** 2024-11-04

**Authors:** Ataru Igarashi, Cillian Copeland, Nikos Kotsopoulos, Riku Ota, Silvia Capucci, Daisuke Adachi

**Affiliations:** 1 Yokohama City University & University of Tokyo, Tokyo, Japan; 2 Global Market Access Solutions Sarl, Chardonne, Switzerland; 3 Novo Nordisk Pharma Ltd. & Novo Nordisk A/S; 4 Novo Nordisk Pharma Ltd.; 5 Aarhus University, Department of Economics and Business Economics & The Research Institute of Economy, Trade and Industry

**Keywords:** obesity, disease burden, lost tax, employment, wages, fiscal analysis

## Abstract

**Introduction:** Obesity continues to represent a significant public health concern, with a broad impact from both a health and economic perspective. **Objective:** This analysis assesses the fiscal consequences of overweight and obesity (OAO) in Japan by capturing obesity-attributable lost tax revenue and increased government transfers using a government perspective. **Methods:** The fiscal burden of OAO was estimated using an age-specific prevalence model, which tracked the Japanese population across different body mass index (BMI) categories. The model was populated with fiscal data for Japan, including employment activity and government spending, to calculate tax revenue and transfer costs. A targeted literature review was conducted to identify data estimating the impact of OAO on employment, income, sick leave, retirement, and mortality. These modifiers were applied to Japanese epidemiological and fiscal projections to calculate government tax revenue and spending. The incremental impact of reducing OAO in the general population was subsequently calculated. Results were estimated based on the 2023 Japanese working-age population aged 18 to 70 years. **Results:** The total fiscal burden of OAO in Japan, defined as BMI of at least 25, is estimated at US 13.41billion(¥1925billion),representing0.46.3 billion (¥901 billion) and 1.2billion(¥179billion)indirectandindirecttaxrevenue,respectively,duetoloweremploymentandincomecombinedwithhighersickleave.ExcessOAO−attributablehealthcarecostswere5.4 billion (¥769 billion), while additional pension payment spending of $0.5 billion (¥77 billion) was estimated, due to higher levels of early retirement. **Conclusions:** While the health implications of OAO are well documented, this fiscal analysis demonstrates the significant economic burden of OAO both to the healthcare system and broader government accounts. Policies aimed at reducing population-level obesity have the potential to benefit government accounts through increasing employment and reducing public spending, which can offset the cost of implementing these policies.

## INTRODUCTION

Obesity continues to represent a significant public health challenge globally.^1^ Recent estimates indicate that by 2035, over 4 billion people could be affected by overweight and obesity (OAO; defined as body mass index [BMI] ≥25 kg/m^2^), with an anticipated rise in the levels of obesity (BMI ≥30 kg/m^2^) from 14% of the population in 2020 to 24% in 2035.^2^ Asian countries have seen a significant increase in the rates of OAO, which has coincided with the economic development in recent decades.^3^ While Japan has not experienced the same level of growth in obesity levels as other Asian countries, a 15.3% increase in the rates of OAO was observed from 1990 to 2013.^3^

Obesity and its associated comorbidities, such as diabetes and cardiovascular disease, lead to significant costs for society, with the *World Obesity Atlas 2023* estimating that the cost of OAO will increase to over US $4 trillion of potential income by 2035.^2^ Several studies have explored the medical expenditure associated with OAO in Japan.^4–6^ While there are differences between the healthcare burden for males and females, there is a clear increase in medical costs for individuals living with overweight or obesity.^4^ Obesity also leads to elevated mortality,^7^ and Asians with obesity have been shown to have higher mortality risk than White populations with obesity.^8^ The burden of OAO is not limited to healthcare costs as, similar to many chronic conditions, it can impair work activity and affect the labor market, which subsequently impacts government accounts through reduced tax revenue and increased social welfare payments.^9–11^ These broader economic effects can manifest in different ways. First, individuals living with obesity may incur productivity losses through absenteeism or presenteeism.^12,13^ Moreover, obesity can lead to lower rates of employment and lower income, although the exact magnitude of this effect can vary between males and females.^14^ Finally, obesity can lead to exit from the workforce entirely, either through long-term disability or early retirement.^15,16^ As such, obesity can have a financial impact on governments due to lower tax revenue combined with higher expenditure on social welfare programs.

The objective of this study is to capture the impact of OAO on Japanese government accounts using a fiscal modeling framework.^17,18^ This study uses a governmental perspective to evaluate revenues and transfers resulting from OAO, such as reduced employment rates, lower income, increased healthcare spending, higher disability and early retirement rates, and increased mortality. These elements can all have fiscal effects (ie, impose economic effects on public budgets through lower tax revenue and increased spending for governmental transfers received by individuals such as pensions and disability benefits).

## METHODS

To assess the burden of disease (BoD) of OAO in Japan from a fiscal perspective, a prevalence-based model was developed in Microsoft Excel. A targeted literature review was undertaken to identify model inputs, specifically modifiers linking BMI with economic effects that may result in changes of governmental revenues or costs, also named as fiscal effects. Such effects include employment rate changes, income reductions and increased needs for social welfare and transfers. The full details of the literature review are presented in **Supplemental Appendix S1**. The present analytic framework resembles that of traditional cost-of-illness studies, however, rather than calculating the population attributable fractions (PAF) of obesity-related comorbidities, this analysis focused on calculating the corresponding impact on public accounts (ie, the amount of tax revenue and government expenditure that can be attributed to OAO).^17,18^ While there have been other studies exploring the fiscal impact of obesity,^19^ this is the first study focusing on the Japanese perspective. As this analysis utilizes data that have been previously published and does not include use of any individual patient-level data, no ethics approval was required.

The model was developed to track the Japanese population across different BMI categories, using data from the Japan National Health and Nutrition Survey.^20^ These data were utilized to form a “control” cohort mirroring the general Japanese population. Fiscal flows of tax revenues and transfer costs were compared with a hypothetical “intervention” cohort, where OAO, defined as BMI of at least 25 kg/m^2^, was removed. This cut-off was adopted to reflect the definition of obesity often used in Asian countries. While the World Health Organization (WHO) categorizes obesity based on a BMI of at least 30 kg/m^2^, it is important to consider the ethnicity-specific impact of different BMI levels, particularly in Asian populations.^5,8,21^ Japanese individuals have been shown to demonstrate health effects associated with excess weight at a lower BMI than White populations,^8,22^ as well as having higher body fat deposition at a given BMI value.^23,24^ Given these differences, an alternate categorization has been proposed for Japan, with obesity defined as BMI of at least 25 kg/m^2^.^8,21,22^ This approach has been suggested as more appropriate for the Japanese population and explains why the prevalence of obesity in Japan is considered low (based on the WHO classifications), although rates of obesity-related conditions reflect those seen in Western countries.^21^ As such, this analysis considered the fiscal effects of the population with BMI of at least 25 kg/m^2^.

The incremental difference between the tax revenue and government expenditure of the control and “intervention” cohorts represents the BoD of OAO. The BoD was initially calculated for 2023 and subsequently projected for a further 10 years to reflect the impact of increasing obesity rates as well as population changes.^25,26^ The modifiers used to model the effect of different BMI categories on government tax revenue and social welfare expenditure were identified through the targeted literature review. The objective of this search was to identify relevant data in Japan, although data from other countries were explored if Japanese-specific information was unavailable. Details of the inclusion and exclusion criteria informing this search are outlined in **Supplementary Appendix S1**.

The targeted literature review focused on the PubMed database and, following identification of 125 records, 17 articles were screened at full-text and 2 studies were included for extraction.^4,27^ Additional hand searches were subsequently undertaken in Google Scholar and Google to identify further studies of relevance. For these hand searches, some

initial restrictions were relaxed, such as the year of publication and scope country, given that limited Japanese-specific evidence was found in the initial PubMed search. Twelve additional studies were sourced from these hand searches and included for extraction.

To construct the 2 cohorts in this analysis, age- and gender-specific data on the prevalence of the following 4 BMI categories were used to categorize the Japanese population^20^:

- Underweight (BMI <18.5)
- Normal weight (BMI 18.5 to < 25)
- Overweight (BMI 25 to <30)
- Obesity (BMI ≥30)

While this analysis is focused on the Japanese categorization of obesity (BMI ≥25), many studies and data sources report results according to the WHO classification of obesity. As such, the general population was split into the WHO BMI categories when modeling each fiscal effect. When constructing the hypothetical “intervention” cohort, all individuals with overweight or obesity (representing the Japanese definition of obesity) were reassigned to the normal weight category.

Economic effects of OAO were modeled using the most relevant fiscal data available for Japan, which included data on employment and income as well as government expenditure on pension payments, sick leave, and health care. Employment and income effects were calculated using results of an observational study investigating the links between OAO and the labor market, which reported the effect of BMI on both the probability of employment and monthly earnings.^14^ Several identified studies also demonstrated the association between OAO and excess healthcare costs, sick leave, and early retirement.^4,16,28^ The underlying calculations used in the analysis are described in **Equations 1-11**. Modifiers identified in the targeted literature review were primarily in the form of regression coefficients for individuals with OAO and hazard ratios (HRs). These values were used to adjust Japanese population-level data to generate relative effects for the OAO group. Employment rates, recipients of social welfare payments, and annual income were calculated using **Equations 1-3**. In **Equation 1**, the published coefficient for OAO was used to adjust the age-specific probability of employment in the general population.^14^ Similarly, population rates of sick leave and retirement were adjusted by the published hazard ratios in **Equation 2**.^16,28^ **Equation 3** calculates adjusted income for OAO individuals using the marginal effect of OAO on income from the published logistic regression identified in the literature search.^14^

**Figure:**
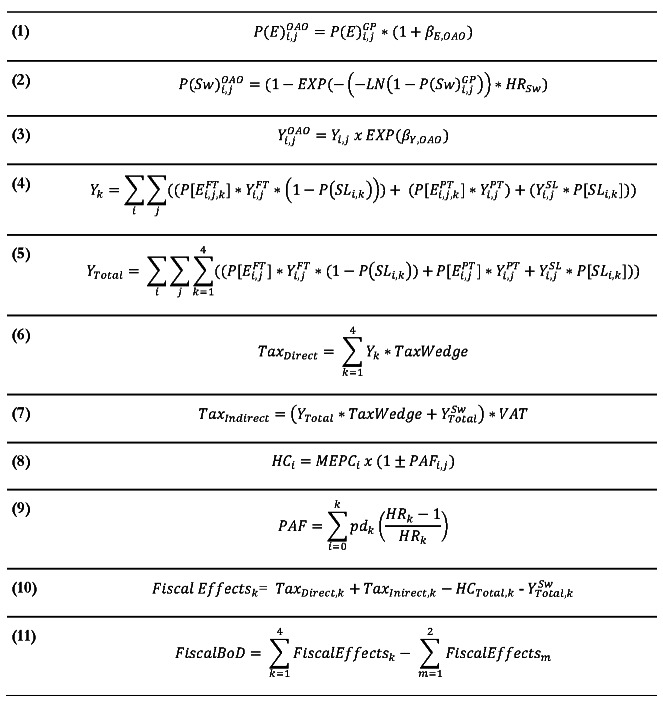
Equations Used in the Analysis For BMI category *k* = 1-4 in the control cohort (ie, the general population) and *m* = 1-2 for the “intervention” cohort where all individual in the overweight and obesity categories are reassigned to the normal weight group (therefore, $\sum_{k=1}^{4}X_{k}=\sum_{m=1}^{2}X_{m}=1$ where *X_k_* and *X_m_* are the proportion of the population in each BMI category). Abbreviations: β, marginal effect from relevant regression model; *GP*, general population; *HC*, annual healthcare costs; *HR*, hazard ratio; *I*, age group; *j*, gender; *MEPC*, medical expenditure per capita; *OAO*, overweight/obesity group; *PAF*, population-attributable fraction; *Pd^k^* proportion of cases in *k*^th^ BMI category; *P(E)*, probability of employment (full-time and part-time); *SL*, sick leave; *Sw*, social welfare (ie, sick leave or retirement); *VAT*, value- added tax; *Y*, employment income.

Total income for each of the 4 BMI categories was calculated by combining those in full-time employment (excluding those on sick leave), those in part-time employment, and those on sick leave (who are assumed to receive a portion of their salary), as outlined in **Equations 4 and 5**. It was conservatively assumed that sick leave would only apply to full-time employees, although a scenario explored the impact of applying the sick leave effect to both full-time and part-time employees. Direct tax revenue was then calculated by applying the Japanese tax wedge to total income (**Equation 6**). The tax wedge represents the ratio of taxes paid by workers including employer labor costs, and are typically reported by the Organization for Economic Co-operation and Development (OECD).^29^ Indirect tax was also captured to reflect government income from consumption taxes (ie, value-added tax [VAT]). The Japanese VAT rate of 10% was applied to disposable income (ie, income following application of the tax wedge) and income from government transfers (ie, pension payments) using **Equation 7**.

Healthcare costs were calculated by combining age-adjusted medical expenditure per capita from the Japanese Ministry of Health, Labor and Welfare with OAO-attributable fractions of healthcare costs from Fujita et al.^4^ For the OAO BMI categories, medical expenditure per capita was adjusted upward with the attributable fractions and downward for the remaining categories (**Equation 8**).

A key element of this analysis is accounting for the mortality impact associated with higher BMI levels. Excess deaths due to OAO were estimated using the PAF formula from Rockhill et al^30^ (**Equation 9**). This formula was applied using data from a pooled analysis of 7 Japanese studies assessing the link between BMI and all-cause mortality.^31^ The PAF was then combined with Japanese life tables to estimate the annual number of OAO-specific deaths.^32^

The total fiscal effect for both simulated populations was calculated as the sum of tax revenue (both direct and indirect) minus government spending on social welfare (pension payments) and healthcare costs (**Equation 10**). Finally, the fiscal burden of OAO was calculated as the incremental fiscal effects between the “control” cohort (ie, general population) and the “intervention” cohort where the prevalence of OAO is set to zero (**Equation 11**).

To generate results for future years, population projections were obtained from the National Institute of Population and Social Security Research.^26^ The prevalence of OAO was assumed to increase annually by 1.47% and 0.47% for men and women, respectively, based on results of a forecasting study in South Korea.^25^ These values were obtained by calculating an average annual increase in OAO based on the forecasted prevalence of OAO from 2020 to 2030.^25^ Similarly, a discount rate of 2% was used in line with Japanese guidance,^33^ and wages were assumed to increase by 2% each year based on the reported average increase in year-on-year total cash earnings in Japan.^34^ The base-case analysis modeled the fiscal effects of individuals aged 18 to 70 (ie, individuals born between 1953 and 2005) for an analytic time horizon of 1 year to capture total fiscal effects within the Japanese working-age population.

An overview of key inputs informing the core model equations is provided in **Supplementary Appendix S2**.

### Scenario and Sensitivity Analysis

Scenario analysis was conducted to assess the impact on results of gender, of focusing only on the WHO categorization of obesity (defined as BMI ≥30 kg/m^2^) and of using alternative sources for mortality for the population at least 65 years of age.^35^ Furthermore, the upper age of the analysis was varied to explore the impact of omitting older age categories and focus on the working-age population (ie, birth cohorts of 1958-2005 [18-65 years of age], birth cohorts of 1948-2005 [18-75 years of age]), and all birth cohorts (ie, birth cohorts of 1919-2005 [18-104 years of age]). A scenario was also included to explore the impact of applying an OAO sick leave effect to both full-time and part-time employees. One-way sensitivity analysis was conducted to explore the impact of individual variables on the total fiscal effect of OAO.

In addition to the prevalence model, a cohort-based analysis was conducted. Specifically, the lifetime of an individual with OAO was modeled assuming a mean age of 49 years and was compared to a non-OAO peer. This cohort modeling approach assumed that individuals cannot switch obesity status throughout their lifetime. The present value of lifetime fiscal effects was estimated for both arms of the model and compared to assess the, per person, lifetime, fiscal effect of OAO. Furthermore, one-way sensitivity analysis was conducted for the average starting age of the analysis.

## RESULTS

The total fiscal burden of OAO in Japan is estimated at ¥1.92 trillion ($13.4 billion) for the year 2023. This comprises a loss of ¥901 billion ($6.3 billion) in direct tax revenue stemming from reduced employment rates and lower income. Indirect tax revenue is also reduced by ¥179 billion ($1.2 billion) as individuals with lower employment and/or income have reduced disposable income which subsequently impacts consumption tax, although there is also a minor increase in indirect tax from higher pension payments (as individuals receiving pension payments will spend part of this income on consumption). Government expenditure on pension payments and healthcare costs increased by ¥77 billion ($0.5 billion) and ¥769 billion ($5.4 billion), respectively. **Table 1** outlines the different categories comprising the total fiscal burden of OAO.

**Table 1.** Fiscal Burden of OAO in Japan (2023)

	**Billion** ¥
Tax revenue	
Direct tax losses from employment	901.23
Indirect tax losses (from employment income and transfers)	178.65
Transfer payments	
Retirement payments	76.80
Healthcare costs	768.58
Total fiscal burden of OAO	1925.26

Abbreviation: OAO, overweight and obesity.

**Figure 1.**
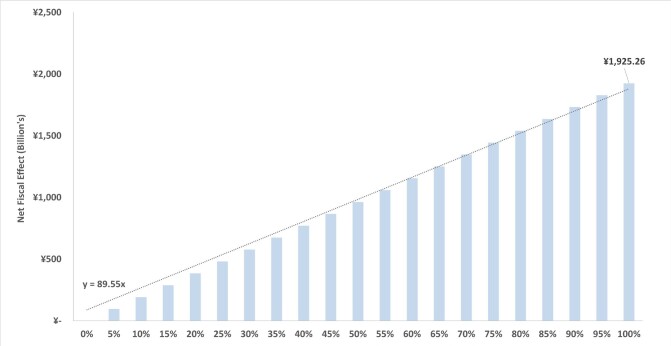
Relationship Between Obesity and Overweight Prevalence Reduction and Net Fiscal Effects (2023)

The total fiscal BoD of OAO represents approximately 0.4% of the gross domestic product (GDP) of Japan (based on 2022 GDP).^36^ Based on demographic and epidemiologic projections the fiscal burden of OAO is expected to increase by 15% in the next decade, with the projected fiscal burden estimated at ¥2223 billion in 2033. Furthermore, this analysis shows that, for each 1% reduction in the prevalence of OAO, there will be a net fiscal gain of ¥89.6 billion for the Japanese government (**Figure 1**).

**Figure 2.**
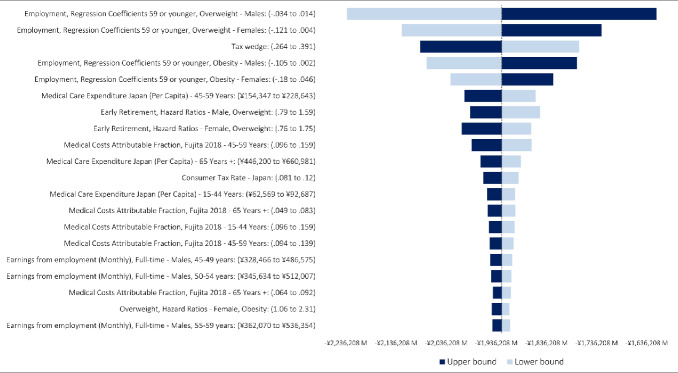
Results of One-way Sensitivity Analysis for the Fiscal Burden of Obesity and Overweight

The tornado diagram generated following the one-way sensitivity analysis is illustrated in **Figure 2** and shows that results are most sensitive to the employment modifiers applied to generate rates for the population with OAO as well as the tax wedge used to calculate direct tax revenue from employment income.

**Table 2** shows the results of the scenarios explored to test the impact of results when different sources and assumptions are applied.

**Table 2.** Scenario Analysis Results (2023)

**Scenario**	**Tax Revenue Loss (Billion ¥)**	**Transfer Spending (Billion ¥))**	**Net Fiscal Effect of OAO (Billion ¥)**
Prevalence model results			
Base case (birth cohorts 1953-2005, 18-70 y)	1079.88	845.38	1925.26
1958-2005 birth cohorts (18-65 y)	1039.09	627.52	1666.62
1948-2005 birth cohorts (18-75 y)	1091.27	1126.12	2217.38
1919-2005 birth cohorts (18-104 y)	1112.93	1550.57	2663.50
Obesity defined as BMI ≥30 kg/m2	715.13	125.57	840.70
Alternate mortality data (≥65 y)	1078.99	846.32	1925.31
Sick leave applied to all employees (FT and PT)	-1080.79	-845.38	-1926.17
Cohort model results (per person)			
Mean age 49 y, 1974 birth cohort	385 884	434 221	820 104
Mean age 35 y, 1988 birth cohort	520 418	335 458	855 876
Mean age 65 y, 1958 birth cohort	63 806	476 081	539 886
Male birth cohort of 1974 (mean age, 49 y)	498 778	510 610	1 009 388
Female birth cohort of 1974 (mean age, 49 y)	279 388	362 160	641 548

Abbreviations: BMI, body mass index; FT, full-time; OAO, overweight and obesity; PT, part-time.

## DISCUSSION

This study assessed the burden of OAO (defined as BMI ≥25 kg/m^2^) in Japan through the lens of government accounts. This perspective captures the broader economic effects of diseases on individuals’ ability to participate in the labor market and subsequently contribute tax revenue to the government. Within a single year, OAO is estimated to cause a total loss of ¥1080 billion in tax revenue, including both direct tax on employment income as well as indirect consumption tax (ie, VAT). This loss represents approximately 2.6% of all income tax and consumption tax revenue in Japan.^37^ OAO also leads to a greater number of individuals retiring, which in turn requires increased government expenditure of ¥77 billion on pension payments. It is well understood that OAO leads to significant healthcare costs, given the variety of associated comorbidities, and this analysis calculated excess obesity-attributable healthcare costs of ¥769 billion. However, healthcare costs are lower than the total loss in OAO-attributable tax revenue, illustrating the magnitude of these broader fiscal effects. Considering the size of this fiscal burden, policies aimed at reducing the prevalence of OAO can lead to substantial savings. Indeed, for every 1% reduction in the prevalence of OAO, the Japanese government will incur a net fiscal effect (combining increased taxes with lower expenditure) of ¥89.6 billion.

Projecting results over a 10-year period illustrates that increasing rates of OAO will ultimately translate to a greater burden falling on the Japanese government, with larger numbers of individuals leaving employment and the workforce as well as increased spending on pension payments and healthcare.

This analysis has several limitations. First, the fiscal effects of obesity have been generated using data from a combination of different observational studies. The economic effect of obesity is well established, with multiple studies illustrating the link between obesity and fiscal effects such as employment, sick leave, early retirement, and general workforce participation.^12,15,38–40^ However, there is some uncertainty regarding the exact magnitude of these effects, as studies often adopt different methodologies when capturing economic effects.^40^ Some economic studies focus on capturing the burden of obesity through individual comorbidities, however, it is difficult to isolate the effects of a comorbidity specifically attributable to obesity vs the comorbidity itself.^40^ There is also uncertainty regarding the additive or multiplicative effect of having different or multiple obesity-related comorbidities and how this would impact labor market outcomes. Due to these challenges, this analysis focused on the links between BMI categories and economic outcomes, as these are more prevalent in the literature. The output from these observational studies was primarily focused on single points in time and provided limited data on the impact of OAO on wages and employment over time. As such, fiscal projections were limited to 10 years, as longer time periods could introduce significant uncertainty to results. The limitation of the primary data source is that it is aggregated data without individual information. Therefore, it was not possible to analyze employment and health outcomes in relation to certain baseline demographic variables, such as age.

The literature search used to identify studies linking OAO to different fiscal outcomes focused initially on Japan, however, as insufficient Japanese studies were found, the search was subsequently broadened to consider international data. While the base-case analysis primarily comprised Japanese data, some data from Korea were utilized, specifically, OAO prevalence projections and the data demonstrating the impact of OAO on early retirement. It is not expected that use of Korean data will significantly skew results, as the prevalence projections are broadly aligned with historical trends in OAO prevalence in Japan,^41^ while the Korean study linking OAO with retirement is not a significant driver of model results.^16^

An additional limitation of this analysis is the use of BMI as the measure of obesity. Some studies have suggested that BMI in isolation is not a fully appropriate way to categorize individuals with obesity, and other measures, such as waist circumference, may be a more appropriate categorization.^42^ However, as BMI has historically been the most common method used to categorize obesity, there is significantly more data available from both published literature and national databases relating to BMI than for other measures.

While this analysis assesses the burden of OAO through a government analytic perspective, the general economic burden of obesity has been explored widely within published literature, both globally and within Japan.^12,40,43,44^ These studies include a range of costs associated with obesity including both medical costs and indirect costs, which can include productivity loss, travel costs, and the cost of informal caregiving.^45^ Some studies also focus on the burden of OAO through BMI categories,^12,19^ while others consider the specific impact of obesity-related comorbidities.^46^ The burden of OAO in Japan has been investigated previously as part of a global BoD study, with the total burden of OAO estimated as 0.99% of Japan’s GDP.^12^ This value is notably higher than the estimate of 0.4% found in this analysis; however, this is likely due to the published study adopting a broader societal perspective than the stricter government perspective.

This analysis focuses on the burden of OAO within the working age population over a 1- to 10-year period and does not consider the potential long-term effects of OAO within the population. Notably, the long-term burden of OAO in adolescents is not considered; however, there would likely be a substantial fiscal burden from increased rates of childhood/adolescent OAO over time as these individuals transition into the workforce. Several studies have investigated the lifetime costs of childhood/adolescent OAO and demonstrate the long-term effects on both direct and indirect costs.^47,48^ Interventions aimed at reducing childhood/adolescent OAO are likely to generate even higher fiscal gains than shown in this analysis, as the benefits will persist for individuals’ entire time as part of the working population. It is unsurprising, therefore, that published studies assessing interventions for childhood OAO have demonstrated a high long-term return on investment.^49^

Given the challenges of increasing healthcare budgets and effectively allocating resources, fiscal analyses can represent a useful tool for governments to understand the cross-sectoral impact of new policies. By evaluating policies within the context of the broader economy, governments can more accurately capture the net effect on public accounts. This in turn can facilitate more holistic and sustainable decision making. The Global Health Strategy of Japan highlights the need for sustainable health financing systems,^50^ and use of a government perspective can help ensure policies are focused on generating the greatest benefit for the economy as a whole, rather than considering individual departments or sectors in isolation.

## CONCLUSION

This study demonstrates the significant impact of OAO on tax revenue and transfer spending using a broad government perspective. This framework captures the effects of OAO within the broader Japanese economy and illustrates how the burden of OAO is not confined to the healthcare system alone. Fiscal analyses provide a holistic perspective on the BoD and can represent a valuable tool for governments when evaluating new policy decisions.

### Disclosures

S.C. and R.O. are employees of Novo Nordisk Pharma Ltd. C.C. and N.K. have received funding from Novo Nordisk Pharma Ltd for their contributions. A.I. is an employee of the University of Tokyo Graduate School of Pharmaceutical Sciences and has received consulting fees from Pfizer Inc., Takeda Pharmaceuticals Inc., and Shionogi Inc.; honoraria from Shionogi Inc.; payment for expert testimony from Pfizer Inc. and Takeda Pharmaceuticals Inc; and has other financial or non-financial interests in Takeda Pharmaceuticals Inc.

## Supplementary Material

Online Supplementary Material
