# Supplementary material for: Assessing the Fiscal Burden of Overweight and Obesity in Japan through Application of a Public Economic Framework
Source: J Health Econ Outcomes Res. 2024 Nov 4;11(2):125–32. doi: 10.36469/001c.123991 (PMC11555824; doi:10.36469/001c.123991)
Supplement: Online Supplementary Material [file jheor_2024_11_2_123991_252000.pdf]

Journal of Health Economics and Outcomes Research

Online Supplementary Material

Assessing the Fiscal Burden of Overweight and Obesity in Japan Through Application of a Public Economic Framework. *JHEOR*. 2024;11(2):125-132. doi:10.36469/jheor.2024.123991

Supplementary Appendix S1: Targeted Literature Review

Table 1: Literature Review Search Strategy

Table 2: PICOS Criteria

Figure 1: PRISMA Diagram: Literature Search Results

Supplementary Appendix S2: Overview of Model Inputs

Table 3: Overview of Key Model Inputs

This supplementary material has been provided by the authors to give readers additional information about their work.

This is an open-access article distributed under the terms of the Creative Commons Attribution 4.0 International License (CCBY-4.0). View this license's legal deed at http://creativecommons.org/licenses/by/4.0 and legal code at http://creativecommons.org/licenses/by/4.0/legalcode for more information.
